# Time until onset of acute kidney injury by combination therapy with “Triple Whammy” drugs obtained from Japanese Adverse Drug Event Report database

**DOI:** 10.1371/journal.pone.0263682

**Published:** 2022-02-09

**Authors:** Yuki Kunitsu, Daiki Hira, Aya Morikochi, Tomohiro Ueda, Tetsuichiro Isono, Shin-ya Morita, Tomohiro Terada

**Affiliations:** 1 Department of Pharmacy, Shiga University of Medical Science Hospital, Otsu, Shiga, Japan; 2 Department of Clinical Pharmacology and Therapeutics, Kyoto University Hospital, Sakyo-ku, Kyoto-shi, Kyoto, Japan; 3 College of Pharmaceutical Sciences, Ritsumeikan University, Kusatsu, Shiga, Japan; University of Sao Paulo Medical School, BRAZIL

## Abstract

Acute kidney injury (AKI) associated with “Triple Whammy” drug therapy consisting of renin-angiotensin system inhibitors, diuretics, and nonsteroidal anti-inflammatory drugs (NSAIDs) has been reported. There have been no reports investigating “Triple Whammy” drug therapy and the time to AKI onset using adverse drug events report databases. The aim of this study was to determine the relationship between the time to AKI onset and treatment with “Triple Whammy” drug therapy. We analyzed AKI cases registered in the Japanese Adverse Drug Event Report database. The data were analyzed using the Kaplan–Meier approach, generalized Wilcoxon tests, and Weibull distribution. AKI was reported in 18,415 cases, of which 7,466 cases used Triple Whammy drugs. All combinations of Triple Whammy drugs were associated with significantly higher odds ratios for reporting AKI. In Weibull analysis, AKI onset was early for most combination patterns of Triple Whammy drugs. The Kaplan–Meier approach showed that the treatment duration to AKI onset was much shorter in cases using NSAIDs; median onsets, 8 days for triple combination, 7 days for NSAIDs added to renin-angiotensin system inhibitors, 9 days for NSAIDs added to diuretics, 6 days for diuretics added to NSAIDs, and 9 days for NSAIDs alone. AKI associated with Triple Whammy drugs is likely to occur in the early stages of treatment, especially with concomitant NSAIDs. Patients should be monitored for the occurrence of AKI within the first 2 weeks.

## Introduction

Several recent studies reported the risk of acute kidney injury (AKI) associated with the combination of renin-angiotensin-system inhibitors (RASIs), including angiotensin-converting enzyme inhibitors (ACEIs) and angiotensin receptor blockers (ARBs), diuretics, and nonsteroidal anti-inflammatory drugs (NSAIDs). This combination of drugs is sometimes referred to as the “Triple Whammy” (TW) [1–6]. Lapi et al. reported that this triple therapy is associated with an increased rate of AKI (rate ratio 1.31 [95% confidence interval (CI): 1.12–1.53]) compared with the combination of ACEIs and ARBs with diuretics. In addition, the highest risk was observed in the first 30 days of use (rate ratio 1.82 [95%CI: 1.35–2.46]) [4]. Regulatory authorities in Australia and New Zealand already recommend avoiding TW. In 2018, the Ministry of Health, Labor and Welfare advocated against the use of this triple therapy in elderly patients. Thus, the risk of AKI due to TW is well known; however, the timing from beginning drug administration to the onset of AKI has not been assessed. Jean et al. demonstrated an association between TW and AKI using the French pharmacovigilance database [7]. This report was based on adverse drug events (ADEs) over 3 years and showed that the reporting odds ratio (ROR) for adverse events for the three drug types was significantly higher than the ROR for one or two of the drugs. However, the variety of combination therapy and the duration until ADEs was not investigated due to the limited number of AKI cases (n = 837). This information is necessary for informing patients and for the early detection of ADEs. Thus, larger databases need to be analyzed to obtain this information.

ADE report databases for pharmacovigilance are maintained in several countries and regions and have been used in many pharmacoepidemiological investigations into specific ADEs. In this study, we analyzed the time between the start of TW drug administration and the onset of AKI using the Japanese Adverse Drug Event Report (JADER) database of the Pharmaceuticals and Medical Devices Agency (PMDA) in Japan.

## Materials and methods

### Data sources

We collected data from the JADER database, an anonymized and aggregated database of ADE reports submitted to the PMDA in Japan [8]. The data released in December 2020, which reported data between April 2004 and August 2020, was used in the present study. This database consisted of four datasets: “DEMO” (patient demographic information), “DRUG” (drug information), “REAC” (ADE information), and “HIST” (medical history).

In the “DEMO” table, age information was classified into age groups (e.g., 10s, 20s, etc.). Cases with incomplete information on sex and age were excluded from the analysis. Patients younger than 20 years old were also excluded from the analysis because NSAIDs are generally not administered to children in Japan. In the “REAC” table, ADEs were coded using “Preferred Terms” (PTs) in the Medical Dictionary for Regulatory Activities (MedDRA). The Japanese version of MedDRA, MedDRA/J ver. 23.1, was used for this analysis. AKI events were identified using PTs related to AKI [9, 10] in the Standardized MedDRA Query for “acute renal failure.” The PTs used for identification of AKI events are shown in S1 Table. The outcomes of ADEs were reported as “Recovered”, “Remission”, “Unrecovered”, “Death”, and “With sequelae”. In the “DRUG” table, drugs were classified as contributing to the ADEs using the terms “suspect drug”, “concomitant drug” or “interaction” for drugs suspected of being associated with the ADEs, another drug used at the time as the ADEs, and drugs suspected of causing drug-drug interactions, respectively. To evaluate the effects of concomitant medications and interactions, all categorized drugs were included in this study.

### Definitions of target and related drugs

“RASIs,” “Diuretics,” and “NSAIDs” were defined according to the drug names listed in the Kyoto Encyclopedia of Genes and Genomes (KEGG) drug database, referring to the Anatomical Therapeutic Chemical Classification System [11–13]. “RASIs” were drugs classified as renin-angiotensin system inhibitors by the KEGG DGROUP. “Diuretics” were drugs classified as loop diuretics, thiazide and thiazide related diuretics, potassium-sparing diuretics, and arginine vasopressin receptor two antagonists by the KEGG DGROUP. “NSAIDs” were drugs classified as nonsteroidal anti-inflammatory drugs by the KEGG DGROUP. Although aspirin was defined as a nonsteroidal anti-inflammatory drug by the KEGG DGROUP, it was not classified as an “NSAID” in this study because it was usually administered in low doses as an antiplatelet drug. Although the adverse effects of cyclooxygenase-2 (COX-2) inhibitors on renal function are still controversial [3, 14–16], we included them as NSAIDs. The drugs defined as “RASIs,” “diuretics,” and “NSAIDs” are shown in S2 Table; together these were defined as “TW drugs.” However, cases with only topical use of NSAIDs were classified as NSAIDs-naive cases.

To avoid confounding causes of AKI by drugs that are not part of the TW combination, the following drugs were defined as AKI-risk drugs based on previous studies [17–20]: valaciclovir hydrochloride, eldecalcitol, edaravone, aciclovir, tazobactam-piperacillin hydrate, vancomycin hydrochloride, famotidine, levofloxacin, proton pump inhibitors (esomeprazole, lansoprazole, omeprazole, pantoprazole, rabeprazole, and vonoprazan), and aminoglycosides (amikacin, arbekacin, bekanamycin, dibekacin, fradiomycin, gentamicin, isepamicin, kanamycin, micronomicin, netilmicin, paromomycin, ribostamycin, streptomycin, and tobramycin).

### AKI signal analysis

We extracted the following data from the JADER database for cases reporting AKI and other ADEs: sex (male), use of TW drugs, use of AKI risk drugs, and elderly age (≥70 years old). Use of TW drugs indicated the use of RASIs, diuretics, and/or NSAIDs. A cross-tabulation table was created for each item. Using the cross-tabulation table, RORs for each drug separately or in combination were calculated using Eq 1.


$$ROR=\frac{odds\;of\;AKI\;reports\;in\;index\;group}{odds\;of\;AKI\;reports\;in\;reference\;group}$$ (1)

The ROR is defined as the odds of a specific adverse effect caused by a specific drug divided by that of other drugs in the database and is frequently used as a relative risk measure for ADEs in spontaneous reporting databases. If the lower limit of the 95% CI for the ROR is greater than 1, a signal is present [21]. A cross-tabulation table was also prepared for cases with reported AKI and other ADEs by concomitant patterns of RASIs, diuretics, and NSAIDs. Based on the table, adjusted RORs were calculated using logistic regression analysis. The following AKI risk factors were covariates in the logistic regression analysis: sex (male) [22, 23] elderly (≥70 years old) [23–25], and AKI risk drugs [17]. In addition, the reporting year was also used as a covariate to address the possibility of reporting bias [10]. Categorical variables were compared using chi-square tests.

### Analysis of duration using TW drugs until AKI

Only cases in which the AKI onset date and TW drug start date were available were included in the study. TW duration between the day of the last TW drug start and AKI onset was calculated for each case (S1 Fig). If AKI occurred on the day of the last TW drug start, the duration was set to 0 days. The Weibull distribution was fitted to the cumulative incidence plots for each pattern of TW drugs, and the shape parameter β was calculated. The Weibull shape parameter test is used for the statistical analysis of time-to-onset data and can describe the varying incidence of adverse events (i.e., changes in risk over time) [26]. The shape parameter β indicates the change in hazard with time. A β value of 1 indicates random failure, in which the hazard is constant regardless of time. A β value of less than 1 indicates early failure, in which the hazard is high early on and the failure rate decreases thereafter. A β value of greater than 1 indicates wear-out failure, in which the hazard increases with time [26–28]. In addition, the cumulative incidence was calculated using the Kaplan–Meier approach and compared with the generalized Wilcoxon test.

Analyses applying the Weibull regression analyses were performed using SAS JMP16^®^ (SAS Institute Inc., Cary, NC, USA) and other statistical analyses were performed using IBM SPSS^®^ version 25 (IBM Corp., Armonk, NY, USA). The significance level was set at 0.05.

### Ethics statement

This study was conducted in accordance with the “Declaration of Helsinki” and approved by the Research Review Board of Shiga University of Medical Science (Approval Number: RRB20-014). The JADER Databases used in this study are existing databases that have been de-linked and anonymized by the PMDA after data acquisition. This database is widely available and does not correspond to personal information. The database is an accumulation of valuable case data reported by medical institutions, and the analysis was conducted in compliance with the terms of use indicated by PMDA and with ethical considerations.

## Results

**Fig 1:**
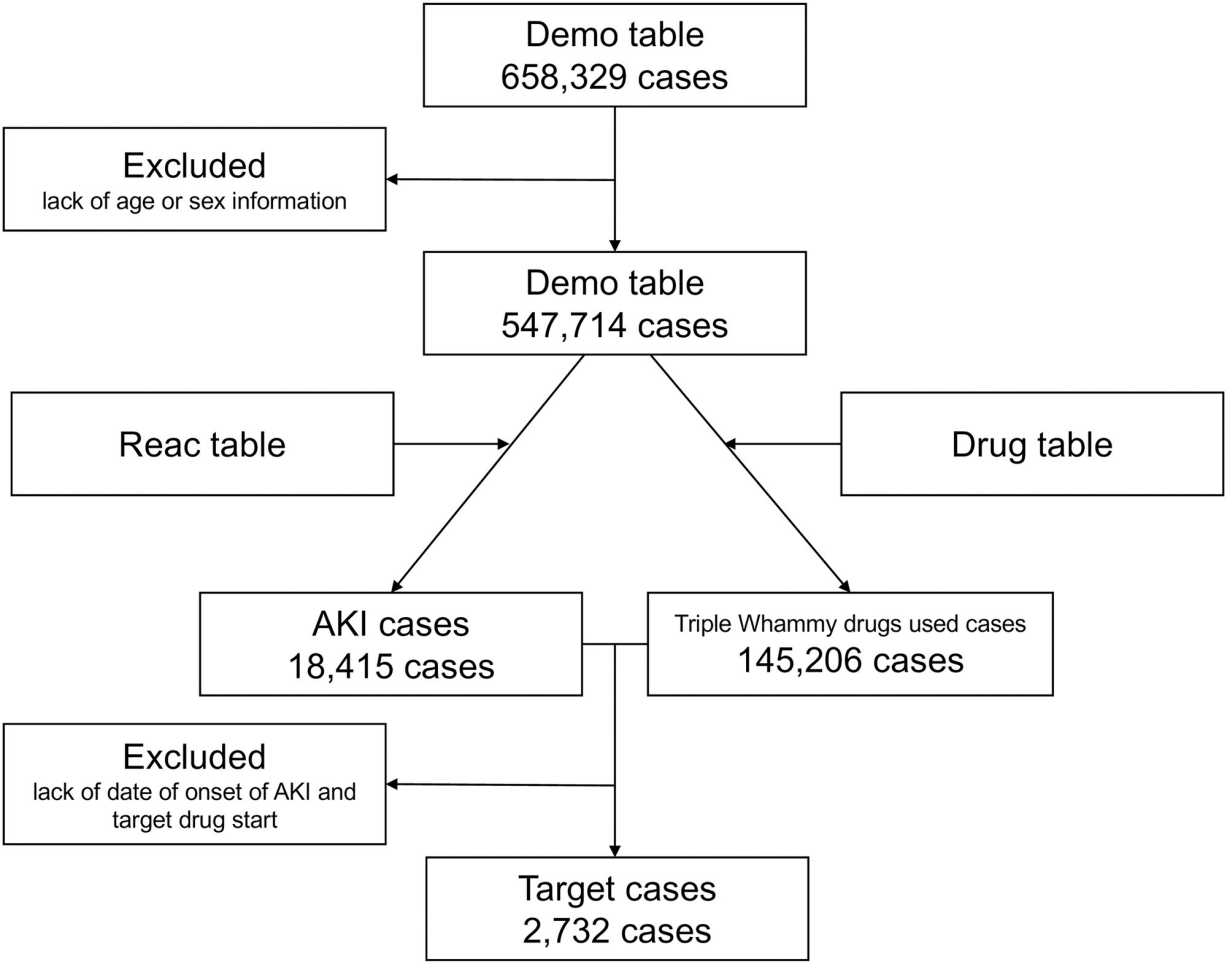
Flow chart of case inclusion and exclusion from the JADER database. AKI, acute kidney injury.

A total of 658,329 ADE cases were obtained from the JADER database (Fig 1). After excluding cases of unknown age or sex, 547,714 cases were used for the analysis. ADEs related to AKI were reported in 18,415 (3.4%) cases, and 145,206 (26.5%) cases used at least one TW drug. AKI with any TW drugs reported in 7,466 cases. After excluding cases of unknown date of onset of AKI and TW drugs start, 2732 cases were used for the analysis of time to onset.

Characteristics of AKI cases and other ADEs are shown in S3 Table. Cases with any of the following factors had significantly higher AKI than other ADEs cases (*P* < 0.001): sex (males), elderly (≥70 years old), and use of any TW drugs or AKI risk drugs. Comparison of TW drug combinations in cases with AKI and other adverse effects are shown in Table 1. Adjusted RORs for single drug groups, double drug groups, and triple drug groups versus no TW drug were 1.50 [95% CI: 1.45–1.56], 2.39 [95% CI: 2.27–2.51], and 2.82 [95% CI: 2.48–3.21], respectively. The outcomes of AKI according to the combination of TW drugs are shown in S4 Table.

**Table 1:** Comparison of TW drug combination in cases with AKI and other ADEs.

RASIs	Diuretics	NSAIDs	Cases with AKI,	Cases with Other ADEs,	Adjusted* ROR [95% CI]
			n (%), n = 18,415	n (%), n = 529,299	(vs. cases without “TW drugs”)
No					
-	-	-	10,949 (59.5)	391,559 (74.0)	(control)
Single					
+	-	-	1,850 (10.0)	37,608 (7.1)	1.43 [1.35–1.50]
-	+	-	1,297 (7.0)	19,433 (3.7)	1.76 [1.66–1.88]
-	-	+	1,872 (10.2)	50,151 (9.5)	1.18 [1.12–1.25]
Total			5,019 (27.3)	107,192 (20.3)	1.41 [1.36–1.46]
Double					
+	+	-	1,454 (7.9)	16,600 (3.1)	2.41 [2.27–2.56]
+	-	+	449 (2.4)	7,438 (1.4)	1.55 [1.40–1.71]
-	+	+	269 (1.5)	3,783 (0.7)	1.70 [1.50–1.93]
Total			2,172 (11.8)	27,821 (5.3)	2.13 [2.02–2.24]
Triple					
+	+	+	275 (1.5)	2,727 (0.5)	2.44 [2.14–2.77]

*Adjusted: Age ≥ 70, Male, any AKI risk drugs, Reporting year; Binomial Logistic Regression Analysis; “+/−” indicates the presence or absence of RASIs, diuretics, or NSAIDs. Day 1 was the day the last TW drug was started. Abbreviations: AKI, acute kidney injury; ADEs, adverse drug events; CI, confidence interval; ROR, report odds ratio; NSAIDs, nonsteroidal anti-inflammatory drugs; RASIs, renin angiotensin-system inhibitors; TW, triple whammy.

Table 2 shows the characteristics of all target cases (2,732 cases) in which the start date of the TW drugs and the onset date of AKI were available. The target cases had similar background information compared to the original population. However, there were only 50 target cases in triple drug groups, because only cases reporting all start dates of TW drugs were extracted.

**Table 2:** The characteristics and TW drug combinations in target cases.

			Target Cases, n (%), n = 2,732
Males			1,624 (59.4)
Elderly (age ≥ 70 years old)			1,459 (53.4)
Triple Whammy drugs used			
RASIs			1,173 (42.9)
Diuretics			1,183 (43.3)
NSAIDs			1,111 (40.7)
any Triple Whammy drugs			2,732 (100)
AKI risk drugs used			
Valaciclovir Hydrochloride			204 (7.5)
Eldecalcitol			54 (2.0)
Edaravone			67 (2.5)
Aciclovir			88 (3.2)
Tazobactam-Piperacillin Hydrate			93 (3.4)
Vancomycin Hydrochloride			90 (3.3)
Famotidine			325 (11.9)
Levofloxacin			116 (4.2)
Proton pump inhibitors			777 (28.4)
Aminoglycosides			57 (2.1)
any AKI risk drugs			1430 (52.3)
Triple Whammy drug combination			
RASIs	Diuretics	NSAIDs	
Single			
+	-	-	584 (21.4)
-	+	-	598 (21.9)
-	-	+	865 (31.7)
Total			2,047 (74.9)
Double			
+	+	-	439 (16.1)
+	-	+	100 (3.7)
-	+	+	96 (3.5)
Total			635 (23.2)
Triple			
+	+	+	50 (1.8)

“+/−” indicates the presence or absence of RASIs, diuretics, or NSAIDs. Day 1 was the day the last TW drug was started. Abbreviations: AKI, acute kidney injury; NSAIDs, nonsteroidal anti-inflammatory drugs; RASIs, renin angiotensin-system inhibitors.

The Weibull regression analyses showed that the shape parameters (β) of most combination patterns were less than 1 (Table 3). A β value less than 1 indicates early failure, in which the hazard is high early on and the failure rate decreases thereafter.

**Table 3:** The parameters for the Weibull distribution for AKI cases by TW drugs combination pattern.

RASIs	Diuretics	NSAIDs	n	Shape parameter: β [95%CI]
Single				
+	-	-	584	0.57 [0.53–0.60]
-	+	-	598	0.49 [0.46–0.52]
-	-	+	865	0.49 [0.47–0.52]
Total			2047	0.47 [0.46–0.49]
	Double			
+	+	-	439	0.62 [0.57–0.67]
1st	2nd		127	0.66 [0.58–0.76]
2nd	1st		112	0.68 [0.58–0.79]
+	-	+	100	0.49 [0.41–0.56]
1st		2nd	60	0.48 [0.39–0.57]
2nd		1st	19	0.58 [0.39–0.80]
-	+	+	96	0.48 [0.41–0.55]
	1st	2nd	45	0.44 [0.35–0.54]
	2nd	1st	39	0.65 [0.50–0.82]
Total			635	0.55 [0.52–0.59]
	Triple			
last			6	1.74 [0.63–3.67]
	last		14	0.51 [0.31–0.76]
		last	16	0.65 [0.43–0.90]
	Total		50	0.51 [0.40–0.63]

Cases in which multiple TW drugs were started at the same time were excluded. Abbreviations: CI, confidence interval; NSAIDs, nonsteroidal anti-inflammatory drugs; RASIs, renin angiotensin-system inhibitors.

The cumulative incidences analyzed using the Kaplan–Meier approach and the generalized Wilcoxon test are shown in Figs 2–5 and S5–S8 Tables, respectively. The onset of AKI was significantly earlier in cases with triple combination and single therapy with TW drugs than onset in double combinations (Fig 2 and S5 Table).

**Fig 2:**
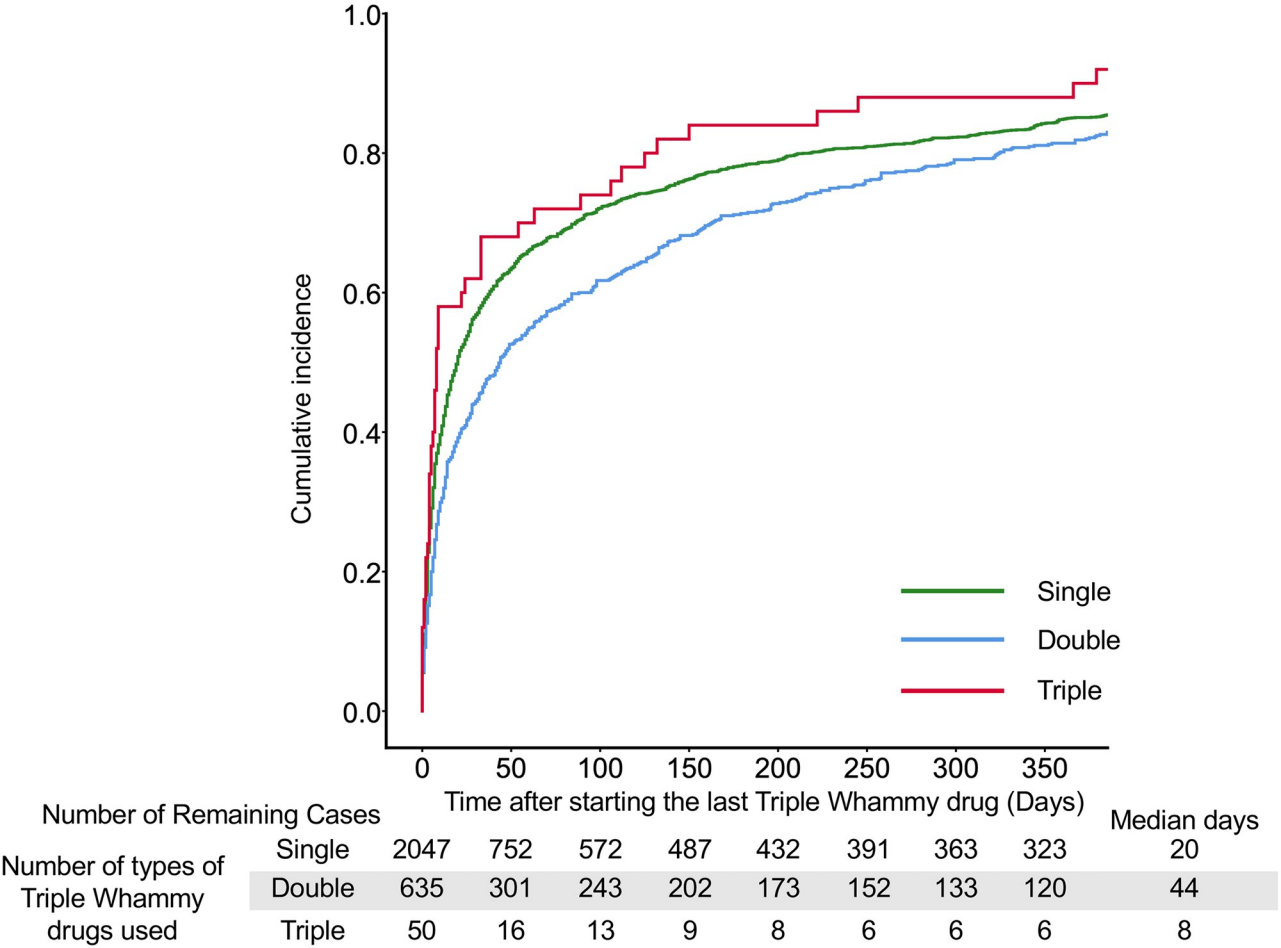
The cumulative incidence with the number of TW drug combinations. Median days indicate the date when the cumulative failure rate reached 50%.

**Fig 3:**
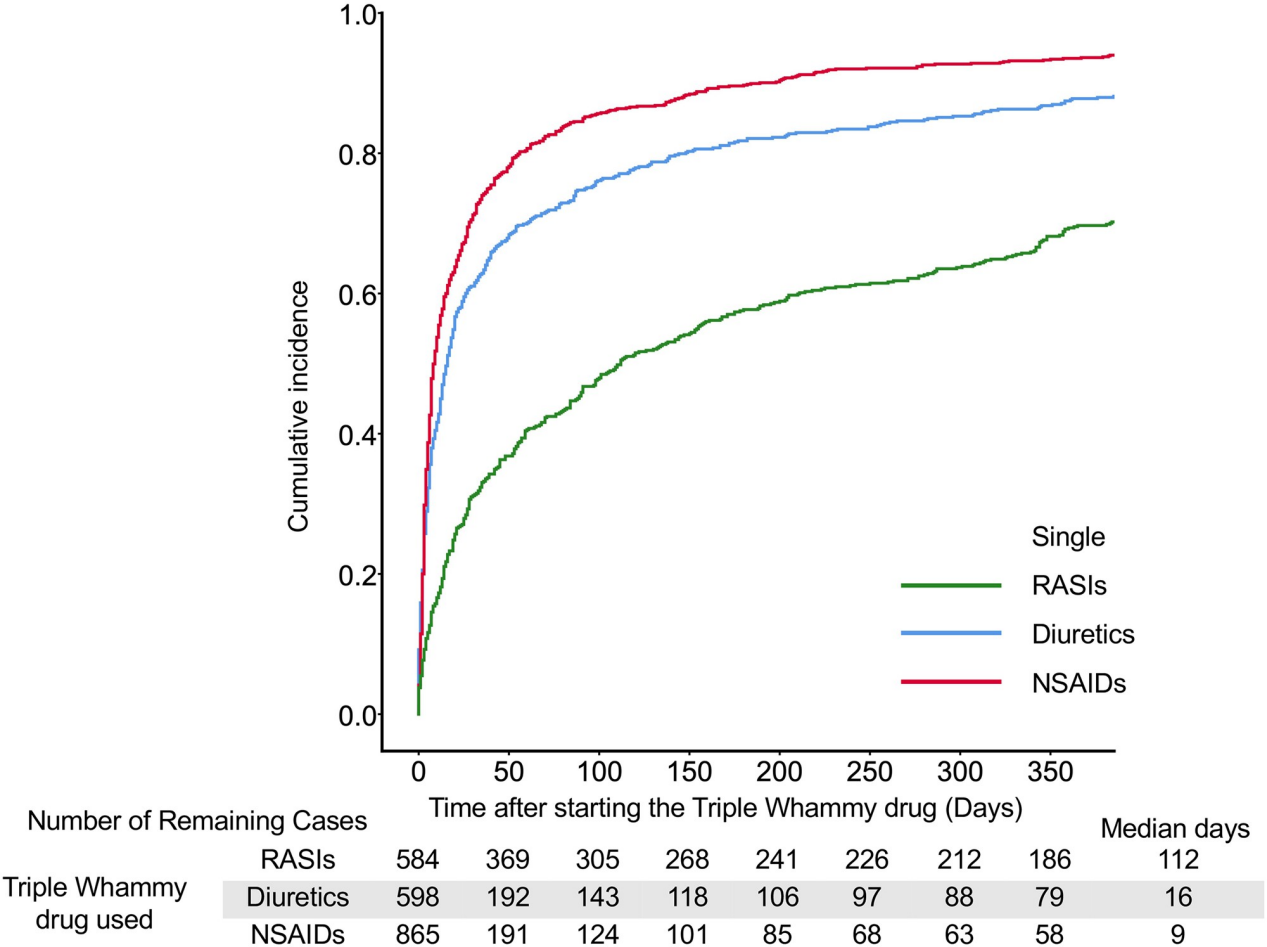
The cumulative incidence with TW drugs in single drug groups. Median days indicate the date when the cumulative failure rate reached 50%.

**Fig 4:**
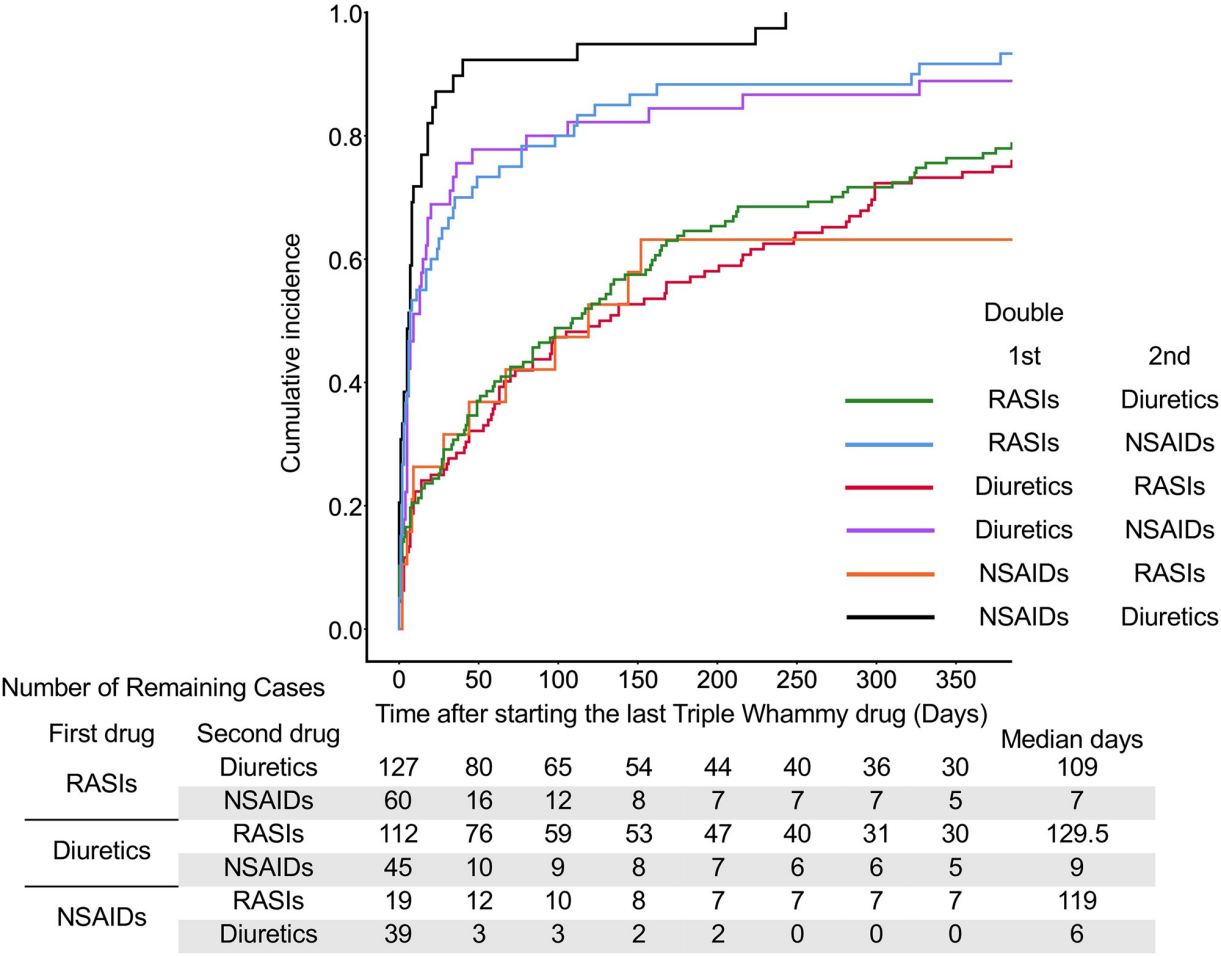
The cumulative incidence with TW drugs used in double drug groups. Cases in which multiple TW drugs were started at the same time were excluded. Median days indicate the date when the cumulative failure rate reached 50%.

**Fig 5:**
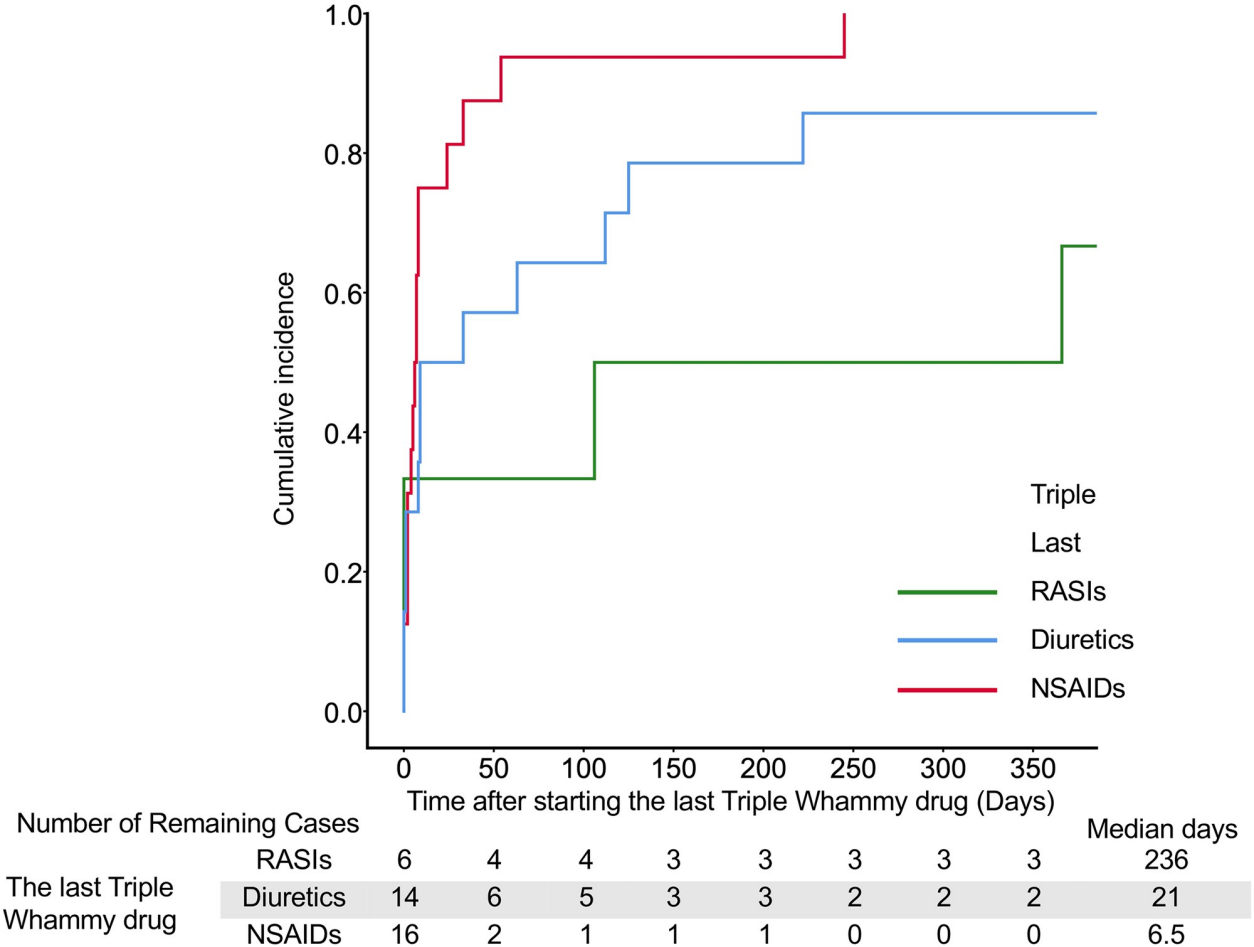
The cumulative incidence with the last TW drugs in the triple drug group. Cases in which multiple TW drugs were started at the same time were excluded. Median days indicate the date when the cumulative failure rate reached 50%.

In single drugs, the onset of AKI was significantly earlier in cases with NSAIDs than the onset in cases with diuretics or RASIs; the median days was 9 days (Fig 3 and S6 Table). In double combinations of TW drugs, the onset of AKI in cases with the addition of diuretics to NSAIDs (median, 6 days), the addition of NSAIDs to RASIs (7 days), and the addition of NSAIDs to diuretics (9 days) were significantly earlier than onsets in the other cases (109–129.5 days) (Fig 4 and S7 Table). There were no significant differences in the onset of AKI among triple combinations of drugs; however, the onset of AKI in cases with the addition of NSAIDs to RASIs and diuretics tended to be earlier than the other combination patterns (Fig 5 and S8 Table).

## Discussion

According to the analysis of the JADER database, the RORs of AKI were significantly higher in all patterns of TW drugs alone or in combination (Table 1). The onset of AKI was an early failure in most TW drug combination patterns (Table 3). The duration until AKI onset was shorter with a median of less than 2 weeks after adding NSAIDs (Figs 3–5).

Several studies demonstrated the risk of AKI associated with the combination of RASIs, diuretics, and NSAIDs [1–6]. However, no reports show the time to onset of AKI or the effects of different drug combinations. In this study, the ROR for AKI was significantly higher for all combination patterns of TW drugs compared to cases with no TW drug; the ROR was adjusted for sex (male), use of AKI risk drugs, elderly (≥70 years old), and reporting year of ADE (Table 1). Based on the French pharmacovigilance database, Jean et al. reported that the number of NSAIDs, RASIs, and/or diuretics, was associated with disproportionate reporting of AKI [7]. Our results also indicated that the RORs for AKI in the ADEs reporting database were different between TW drug combinations. However, in the analysis of the ADEs reporting database, because the number of patients using the target drug and patient background factors are unknown, comparisons using ROR as a direct risk indicator are not appropriate [17]. Therefore, we cannot discuss the synergistic effect of TW drug combinations based on these results, and further studies with a well-defined patient population are needed to assess the true risk of each drug combination pattern.

In Weibull analysis, AKI onset of most combination patterns were early failure types (Table 3). When the last TW drug was an NSAID, the median time to AKI onset was less than 2 weeks (Figs 3–5). On the other hand, the median time to AKI onset was greater than 60 days when RASIs were administered alone or in combination with diuretics (Figs 3 and 4). In the case with the addition of RASIs to NSAIDs, the median time to AKI onset was 119 days (Fig 4). These results suggest a weaker association between RASIs and early AKI onset. Lapi et al. reported that the risk of AKI after initiation of TW drugs combination peaked within the first 30 days and progressively decreased after 30 days [4]. The present study is in agreement with the report by Lapi et al.; however, the present study provides additional information concerning the dependence of AKI onset on the combination pattern of TW drugs (Figs 2–5). When NSAIDs are initiated or added to RASIs or diuretics, patients should be monitored for AKI onset in the first 2 weeks.

RASIs, diuretics, and NSAIDs are risk factors for AKI. Angiotensin-converting enzyme inhibitors and angiotensin II receptor antagonists vasodilate the renal efferent arterioles, resulting in reduced glomerular filtration pressure. During hypovolemia, reduced efferent vascular tone lowers the glomerular filtration rate and ultimately promotes AKI [29]. Diuretics were previously used to treat and prevent AKI [30]; however, diuretics, especially when combined with RASIs and NSAIDs, are now limited because they cause dehydration, which leads to decreased renal blood flow and an increased risk of prerenal AKI [31]. NSAIDs inhibit prostaglandin production via cyclooxygenase inhibition. The inhibition of prostaglandin E2 and prostacyclin synthesis leads to contraction of arteries in the kidney and the resulting decreases in renal blood flow and glomerular filtration rates lead to AKI [32]. In addition, NSAIDs can cause acute tubular necrosis or acute interstitial nephritis [33]. Although the cause for AKI in individual cases was not clear, the early onset of AKI in combination with diuretics and NSAIDs might be due to the synergistic effects of the two drugs, resulting in prerenal AKI with reduced renal blood flow.

There are several limitations to the ADE reporting databases [9, 10, 17]. First, ADE reporting databases only contain information about patients who have experienced ADEs. Therefore, the incidence of ADEs for a target drug cannot be determined [9, 17]. Second, because the database is based on spontaneous reports, reporting bias may exist. For example, underreporting of AKI associated with NSAIDs is likely because renal adverse reactions associated with NSAIDs are already well known [10]. ROR is not a robust indicator of signal strength and may not correspond to the risk of ADEs. Therefore, a direct risk should not be inferred using the RORs. In addition, we may have extracted relatively serious AKI conditions due to the use of billing codes and reporting bias [34]. The outcome information had no details, including temporal information. Therefore, the severity of AKI and the course of treatment could not be assessed. Clinical studies that accurately assess the severity and risk of AKI using certain criteria are needed in the future. Third, analysis of the confounding factors was difficult due to the lack of disease information (e.g., chronic kidney, pulmonary, heart, and hepatic disease, cancer, and diabetes), clinical laboratory values (e.g., serum creatinine and hemoglobin levels), and lifestyle [9, 17]. In addition, some age, sex, and prescription information were missing and cases with missing information were excluded from the analyses. In particular, AKI onset time could be calculated in only 36.6% (2,732/7,466) of cases. The risk of AKI may be different among the TW drugs. Among diuretics, the risk of AKI may be different depending on the mechanism and the strength of the diuretic effect. Among NSAIDs, the effect of selective COX-2 inhibitors on renal function is controversial [3, 14–16]. Bias due to patient conditions was also likely. RASIs are frequently used for hypertensive patients and diuretics are frequently used for hypertensive and heart failure patients; both hypertension and heart failure are risk factors for decreased renal function [35, 36]. Patients use NSAIDs for analgesia or anti-inflammatory purposes, and inflammation is also a risk factor for decreased renal function. Thus, patients who used TW drug combinations overlapped with high-risk conditions for decreased kidney function. Despite these limitations, we could assess the risk of real-world drug combination patterns. Further studies based on populations with known background factors are needed to more accurately assess the increased risk of AKI associated with TW drugs.

## Conclusion

The onset of AKI due to TW drugs was early. In particular, the median time to onset of AKI was less than 2 weeks when the last added TW drugs were NSAIDs.

## Supporting information

S1 Fig Time series of TW drug use and time to AKI onset. (TIF)

S1 Table The Preferred Term (PT) list for identification of AKI events. (PDF)

S2 Table The definition list of TW drugs. Abbreviations: NSAIDs, nonsteroidal anti-inflammatory drugs; RASIs, renin angiotensin-system inhibitors.
(PDF)

S3 Table The characteristics of cases with AKI and other adverse effects. Abbreviations: AKI, acute kidney injury; NSAIDs, nonsteroidal anti-inflammatory drugs; RASIs, renin angiotensin-system inhibitors.
(PDF)

S4 Table The outcome of AKI depending on the combination of TW drugs. Abbreviations: AKI, acute kidney injury; NSAIDs, nonsteroidal anti-inflammatory drugs; RASIs, renin angiotensin-system inhibitors.
(PDF)

S5 Table The generalized Wilcoxon test sorted by the number of TW drug combinations. Cases in which multiple TW drugs were started at the same time were not included.
(PDF)

S6 Table The generalized Wilcoxon test sorted by the TW drug in the single drug group. Cases in which multiple TW drugs were started at the same time were not included. Abbreviations: NSAIDs, nonsteroidal anti-inflammatory drugs; RASIs, renin angiotensin-system inhibitors.
(PDF)

S7 Table The generalized Wilcoxon test sorted by TW drug order in the double drug groups. Cases in which multiple TW drugs were started at the same time were not included. Abbreviations: NSAIDs, nonsteroidal anti-inflammatory drugs; RASIs, renin angiotensin-system inhibitors.
(PDF)

S8 Table The generalized Wilcoxon test sorted by the last TW drug in the triple drug group. Cases in which multiple TW drugs were started at the same time were not included. Abbreviations: NSAIDs, nonsteroidal anti-inflammatory drugs; RASIs, renin angiotensin-system inhibitors.
(PDF)

S9 Table Dataset for time until AKI onset by TW drug combination. Abbreviations: AKI, acute kidney injury; NSAIDs, nonsteroidal anti-inflammatory drugs; RASIs, renin angiotensin-system inhibitors.
(XLSX)
