# Supplementary material for: Time until onset of acute kidney injury by combination therapy with “Triple Whammy” drugs obtained from Japanese Adverse Drug Event Report database
Source: PLoS One. 2022 Feb 9;17(2):e0263682. doi: 10.1371/journal.pone.0263682 (PMC8827454; doi:10.1371/journal.pone.0263682)
Supplement: S1 Table — (PDF) [file pone.0263682.s002.pdf]

S1 Table. The Preferred Term (PT) list for identification of AKI events.

Standard MedDRA Query	PT code	PT
"Acute renal failure" [20000003]	10002847	Anuria
	10003885	Azotaemia
	10018875	Haemodialysis
	10029155	Nephropathy toxic
	10030302	Oliguria
	10034660	Peritoneal dialysis
	10038435	Renal failure
	10038447	Renal failure neonatal
	10049776	Renal impairment neonatal
	10049778	Neonatal anuria
	10053090	Haemofiltration
	10061105	Dialysis
	10062237	Renal impairment
	10066338	Continuous haemodiafiltration
	10069339	Acute kidney injury
	10069688	Acute phosphate nephropathy
	10072370	Prerenal failure
	10078987	Foetal renal impairment
	10081980	Subacute kidney injury
