# Supplementary material for: Time until onset of acute kidney injury by combination therapy with “Triple Whammy” drugs obtained from Japanese Adverse Drug Event Report database
Source: PLoS One. 2022 Feb 9;17(2):e0263682. doi: 10.1371/journal.pone.0263682 (PMC8827454; doi:10.1371/journal.pone.0263682)
Supplement: S2 Table — Abbreviations: NSAIDs, nonsteroidal anti-inflammatory drugs; RASIs, renin angiotensin-system inhibitors. (PDF) [file pone.0263682.s003.pdf]

**S2 Table. The definition list of TW drugs.**

| RASIs | Diuretics | NSAIDs | |
|---|---|---|---|
| (Angiotensin-converting enzyme inhibitors) | (Loop Diuretics) | Acemetacin | Ketophenylbutazone |
| Alacepril | Azosemide | Alminoprofen | Ketoprofen |
| Benazepril Hydrochloride | Bumetanide | Amfenac Sodium Hydrate | Lornoxicam |
| Captopril | Etacrynic Acid | Aminopyrine | Loxoprofen Sodium Hydrate |
| Cilazapril Hydrate | Furosemide | Ampiroxicam | Mefenamic Acid |
| Delapril Hydrochloride | Piretanide | Aspirin | Meloxicam |
| Enalapril Maleate | Torasemide | Bendazac | Mesalazine |
| Imidapril Hydrochloride | (Thiazide and Thiazide related Diuretics) | Benzydamine Hydrochloride | Methyl Salicylate |
| Lisinopril Hydrate | Benzylhydrochlorothiazide | Bromfenac Sodium Hydrate | Migrenin |
| Perindopril Erbumine | Chlortalidone | Bufexamac | Mofezolac |
| Quinapril Hydrochloride | Clofenamide | Celecoxib | Nabumetone |
| Temocapril Hydrochloride | Clorexolone | Choline Salicylate | Naproxen |
| Trandolapril | Hydrochlorothiazide | Diclofenac Sodium | Nepafenac |
| (Angiotensin II receptor antagonists) | Indapamide | Ethenzamide | Nifenazone |
| Azilsartan | Mefruside | Etodolac | Oxaprozin |
| Candesartan Cilexetil | Methyclothiazide | Felbinac | Piroxicam |
| Irbesartan | Meticrane | Felbinac Ethyl | Pranoprofen |
| Losartan Potassium | Trichlormethiazide | Fenbufen | Proglumetacin Maleate |
| Olmesartan Medoxomil | (Potassium-Sparing Diuretics) | Fenoprofen Calcium | Protizinic Acid |
| Telmisartan | Eplerenone | Floctafenine | Salazosulfapyridine |
| Valsartan | Potassium Canrenoate | Flufenamate Aluminum | Salicylamide |
| (Renin inhibitor) | Spironolactone | Flufenamic | Salicylic Acid |
| Aliskiren Fumarate | Triamterene | Flurbiprofen | Sodium Salicylate |
| (Angiotensin receptor-neprilysin inhibitor) | (Vasopressin Receptor 2 antagonists) | Flurbiprofen Axetil | Sulindac |
| Sacubitril Valsartan Sodium Hydrate | Mozavaptan Hydrochloride | Ibuprofen | Sulpyrine Hydrate |
| | Tolvaptan | Ibuprofen L-lysine | Suprofen |
| | | Ibuprofen Piconol | Tenoxicam |
| | | Indometacin | Tiaprofenic Acid |
| | | Indometacin Farnesil | Tolfenamic Acid |
| | | Indometacin Sodium Hydrate | Ufenamate |
| | | Isopropylantipyrine | Zaltoprofen |

Abbreviations: NSAIDS, nonsteroidal antiinflammatory drugs; RASIs, renin angiotensin-system inhibitors.
