# Supplementary material for: Time until onset of acute kidney injury by combination therapy with “Triple Whammy” drugs obtained from Japanese Adverse Drug Event Report database
Source: PLoS One. 2022 Feb 9;17(2):e0263682. doi: 10.1371/journal.pone.0263682 (PMC8827454; doi:10.1371/journal.pone.0263682)
Supplement: S3 Table — Abbreviations: AKI, acute kidney injury; NSAIDs, nonsteroidal anti-inflammatory drugs; RASIs, renin angiotensin-system inhibitors. (PDF) [file pone.0263682.s004.pdf]

S3 Table. The characteristics of cases with AKI and other adverse effects.

	Cases with AKI, n (%), n = 18,415		Cases with Other ADEs, n (%), n = 529,299		p value (chi-square test)
Males	10,606	(57.6)	270,606	(51.1)	<0.001
Elderly (age ≥ 70 years old)	9,749	(52.9)	228,628	(43.2)	<0.001
Triple Whammy drugs used					
RASIs	4,028	(21.9)	64,373	(12.2)	<0.001
Diuretics	3,295	(17.9)	42,543	(8.0)	<0.001
NSAIDs	2,865	(15.6)	64,099	(12.1)	<0.001
any Triple Whammy drugs	7,466	(40.5)	137,740	(26.0)	<0.001
AKI risk drugs used					
Valaciclovir Hydrochloride	1,571	(8.5)	3,458	(0.7)	<0.001
Eldecalcitol	599	(3.3)	4,100	(0.8)	<0.001
Edaravone	290	(1.6)	2,280	(0.4)	<0.001
Aciclovir	525	(2.9)	5,619	(1.1)	<0.001
Tazobactam-Piperacillin Hydrate	468	(2.5)	3,912	(0.7)	<0.001
Vancomycin Hydrochloride	591	(3.2)	4,751	(0.9)	<0.001
Famotidine	1,192	(6.5)	28,655	(5.4)	<0.001
Levofloxacin	526	(2.9)	12,470	(2.4)	<0.001
Proton pump inhibitors	2,967	(16.1)	70,785	(13.4)	<0.001
Aminoglycosides	286	(1.6)	2,641	(0.5)	<0.001
any AKI risk drugs	7,032	(38.2)	117,869	(22.3)	<0.001

Abbreviations: AKI, acute kidney injury; ADEs, adverse drug events; NSAIDs, nonsteroidal antiinflammatory drugs; RASIs, renin angiotensin-system inhibitors.
