# Supplementary material for: Time until onset of acute kidney injury by combination therapy with “Triple Whammy” drugs obtained from Japanese Adverse Drug Event Report database
Source: PLoS One. 2022 Feb 9;17(2):e0263682. doi: 10.1371/journal.pone.0263682 (PMC8827454; doi:10.1371/journal.pone.0263682)
Supplement: S4 Table — Abbreviations: AKI, acute kidney injury; NSAIDs, nonsteroidal anti-inflammatory drugs; RASIs, renin angiotensin-system inhibitors. (PDF) [file pone.0263682.s005.pdf]

S4 Table. The outcome of AKI depending on the combination of TW drugs.

RASIs	Diuretics	NSAIDs	Cases with AKI, n (%), n = 18,415		AKI Outcome, n (%)											
					Recovered		Remission		Unrecovered		With sequelae		Death		Unknown	
	No															
-	-	-	10,949	(59.5)	2,830	(25.8)	2,397	(21.9)	1,336	(12.2)	771	(7.0)	139	(1.3)	3,476	(31.7)
	Single															
+	-	-	1,850	(10.0)	627	(33.9)	531	(28.7)	294	(15.9)	48	(2.6)	79	(4.3)	271	(14.6)
-	+	-	1,297	(7.0)	302	(23.3)	272	(21.0)	259	(20.0)	25	(1.9)	205	(15.8)	234	(18.0)
-	-	+	1,872	(10.2)	671	(35.8)	565	(30.2)	200	(10.7)	36	(1.9)	123	(6.6)	277	(14.8)
	Total		5,019	(27.3)	1,600	(31.9)	1,368	(27.3)	753	(15.0)	109	(2.2)	407	(8.1)	782	(15.6)
	Double															
+	+	-	1,454	(7.9)	442	(30.4)	427	(29.4)	207	(14.2)	39	(2.7)	104	(7.2)	235	(16.2)
+	-	+	449	(2.4)	184	(41.0)	142	(31.6)	42	(9.4)	12	(2.7)	24	(5.3)	45	(10.0)
-	+	+	269	(1.5)	82	(30.5)	70	(26.0)	44	(16.4)	7	(2.6)	40	(14.9)	26	(9.7)
	Total		2,172	(11.8)	708	(32.6)	639	(29.4)	293	(13.5)	58	(2.7)	168	(7.7)	306	(14.1)
	Triple															
+	+	+	275	(1.5)	100	(36.4)	85	(30.9)	37	(13.5)	6	(2.2)	15	(5.5)	32	(11.6)
