# Supplementary material for: Time until onset of acute kidney injury by combination therapy with “Triple Whammy” drugs obtained from Japanese Adverse Drug Event Report database
Source: PLoS One. 2022 Feb 9;17(2):e0263682. doi: 10.1371/journal.pone.0263682 (PMC8827454; doi:10.1371/journal.pone.0263682)
Supplement: S5 Table — Cases in which multiple TW drugs were started at the same time were not included. (PDF) [file pone.0263682.s006.pdf]

S5 Table. The generalized Wilcoxon test sorted by the number of TW drug combinations.

		Number of types of Triple Whammy drug used					
		Single		Double		Triple	
		Chi-square value	p	Chi-square value	p	Chi-square value	p
Number of types of Triple Whammy drug used	Single	-	-				
	Double	24.74	<0.001	-	-		
	Triple	2.66	0.10	11.78	<0.01	-	-

Cases in which multiple TW drugs were started at the same time were not included.
