# Supplementary material for: Time until onset of acute kidney injury by combination therapy with “Triple Whammy” drugs obtained from Japanese Adverse Drug Event Report database
Source: PLoS One. 2022 Feb 9;17(2):e0263682. doi: 10.1371/journal.pone.0263682 (PMC8827454; doi:10.1371/journal.pone.0263682)
Supplement: S6 Table — Cases in which multiple TW drugs were started at the same time were not included. Abbreviations: NSAIDs, nonsteroidal anti-inflammatory drugs; RASIs, renin angiotensin-system inhibitors. (PDF) [file pone.0263682.s007.pdf]

S6 Table. The generalized Wilcoxon test sorted by the TW drug in the single drug group.

		Triple Whammy drug used					
		RASIs		Diuretics		NSAIDs	
		Chi-square value	p	Chi-square value	p	Chi-square value	p
Triple Whammy drug used	RASIs	-	-				
	Diuretics	144.2	<0.001	-	-		
	NSAIDs	275.1	<0.001	12.17	<0.001	-	-

Cases in which multiple TW drugs were started at the same time were not included. Abbreviations: NSAIDs, nonsteroidal antiinflammatory drugs; RASIs, renin angiotensin-system inhibitors.
