# Supplementary material for: Time until onset of acute kidney injury by combination therapy with “Triple Whammy” drugs obtained from Japanese Adverse Drug Event Report database
Source: PLoS One. 2022 Feb 9;17(2):e0263682. doi: 10.1371/journal.pone.0263682 (PMC8827454; doi:10.1371/journal.pone.0263682)
Supplement: S7 Table — Cases in which multiple TW drugs were started at the same time were not included. Abbreviations: NSAIDs, nonsteroidal anti-inflammatory drugs; RASIs, renin angiotensin-system inhibitors. (PDF) [file pone.0263682.s008.pdf]

S7 Table. The generalized Wilcoxon test sorted by TW drug order in the double drug groups.

	First drug	RASIs				Diuretics				NSAIDs			
	Second drug	Diuretics		NSAIDs		RASIs		NSAIDs		RASIs		Diuretics	
First drug	Second drug	Chi-square value	p	Chi-square value	p	Chi-square value	p	Chi-square value	p	Chi-square value	p	Chi-square value	p
RASIs	Diuretics	-	-										
	NSAIDs	22.45	<0.001	-	-								
Diuretics	RASIs	0.62	0.43	29.63	<0.001	-	-						
	NSAIDs	16.97	<0.001	0.17	0.68	21.28	<0.001	-	-				
NSAIDs	RASIs	0.24	0.62	8.13	<0.01	0.01	0.92	4.48	0.01	-	-		
	Diuretics	47.65	<0.001	4.12	0.042	57.84	<0.001	4.82	0.028	16.30	<0.001	-	-

Cases in which multiple TW drugs were started at the same time were not included. Abbreviations: NSAIDs, nonsteroidal antiinflammatory drugs; RASIs, renin angiotensin-system inhibitors.
